# Enhancement of Carbon Nanotube Particle Distribution in PPS/PEEK/Carbon Nanotube Ternary Composites with Sausage-Like Structure

**DOI:** 10.3390/polym8020050

**Published:** 2016-02-16

**Authors:** Lin Cao, Shuling Deng, Zhidan Lin

**Affiliations:** College of Science and Engineering, Jinan University, Guangzhou 510632, China; linc19993@163.com (L.C.); slingdeng@163.com (S.D.)

**Keywords:** PEEK, PPS, thermoplastic composites, MWCNT, processing-structure properties relations, electrical conductive materials, morphology

## Abstract

Carbon nanomaterial particles were selectively distributed in an incompatible and high-melting-temperature polymer blend interface, or in a particular phase, to obtain conductive composites. The composite products revealed poor morphology stability and mechanical performance due to processing several times. Poly(phenylene sulfide) (PPS) and poly(ether ether ketone) (PEEK) polymers with large differences of processing temperatures were selected as blend components to obtain a compatible blend. PPS/PEEK/multi-walled carbon nanotube (MWCNT) ternary nanocomposites were prepared using a controlled melt blending process. The composite samples with similar sausage-like structures of PEEK, as a dispersed phase, promote MWCNT to maximize concentration distribution in the PPS continuous phase. As a result, the theoretical percolation threshold of the composite reduced to 0.347 wt %. Moreover, the conductivity of the composite remained stable even after processing several times. CNTs revealed a particular effect when distributed selectively in this kind of system: it can enhance the dispersion of phases and also provide conductivity to the blend at small CNT contents, which can provide more useful ideas for the development of high-melting-temperature and antistatic or conductive plastic materials.

## 1. Introduction

The preparation of conductive polymer composites through compounding of immiscible and high-melting-temperature polymers containing conductive fillers has attracted much attention in recent years. The outstanding characteristics of conductive polymer composites make them widely popular in various applications, including electronics and electrical appliances, automobiles, and aerospace [1,2,3,4]. Electrical percolation threshold (EPT) is the minimum conductive filler particle content at which a continuous conducting network is formed, resulting in an electrically conductive polymer matrix. To maintain the mechanical and rheological properties, and to decrease the costs, the EPT value of filler in matrix should be decreased [5,6,7,8].

There are several ways to enhance the dispersion and distribution of conductive filler particles in polymer matrices, including using special dispersion techniques, surface treatment of filler particles, and selection of smaller filler particles. Compared with metal conductive fillers, nanoscale carbon materials (such as carbon black, nanodiamonds, graphene, and carbon nanotubes) may provide conductive polymer composites with low density and high electrical conductivity. However, high contents of nanoscale carbon materials in the polymer matrix may adversely affect the rheological and mechanical properties of the composites.

Another important method to reduce the EPT value is to add nanoconductive fillers into incompatible and high-melting-temperature polymer blends, leading to the formation of a continuous structure. Nanoconductive fillers can be selectively distributed in one phase of the polymer blend, thus decreasing the threshold level. In the recent years, there has been a growing interest in the polymer/polymer/nanofiller ternary composites [9,10,11,12]. With the consideration of interactions between nanofiller particles with the two polymers in the blend, three basic structures may exist in which nanofiller particles can be: dispersed in one polymer, dispersed in both polymers, and located at the interface between the two polymers.

Compared with the selective distribution of nanofiller particles in one phase of an immiscible polymer blend, the selective distribution of particles at the interface of two polymers is proposed to be the ideal scenario to achieve the minimum EPT value [13]. During the sample preparation process, the transfer dynamics and the stability of different solid nanofillers at the interface of immiscible polymer blends depend on the particles’ aspect ratio [14]. A larger aspect ratio results in a further migration rate of nanofillers across the interface, reducing the stability of such nanofillers in the interface area. Among the carbon materials, CNTs have larger aspect ratios than graphene, which makes their selective distribution at the interface of immiscible polymer blends very difficult. However, from the structural perspective, CNTs have greater potential for decreasing the electrical resistivity of a polymer blend due to their much larger aspect ratios, which enables the formation of a percolated network at a smaller content compared with carbon black.

However, there are two major problems for carbon materials to enhance their selective distribution in a certain phase of the incompatible and high-melting-temperature blend or at the conductive interface. First, the subsequent molten molding process may partly, or even completely, destroy the co-continuous structure of the blend or the network structure of carbon materials. Second, the mechanical performance cannot meet the requirements of many end applications because of a weak interface in conductive incompatible and high-melting-temperature blends. Therefore, achievement of a conductive blend with a stable conductive network and acceptable mechanical properties deserves academic and industry concern. Based on the difference of melting point between poly(phenylene sulfide) (PPS) and poly(ether ether ketone) (PEEK), their compatibility in the molten state, and separation in the solid phase, we prepared the PPS/PEEK (1:1) blend with a cellular structure via a melt blending method at temperatures above the melting point of PEEK. To obtain a PPS/PEEK/MWCNT high-melting-temperature composite, a PPS/PEEK blend and MWCNT were melt blended at the temperatures between the melting points of PPS and PEEK in which the selective distribution and its pattern for MWCNT particles under compression can be observed. The PEEK with the cellular structure is not only compressible to the space distribution of MWCNT particles, it also has certain enhancement and toughening effects on PPS. Processing of PPS/PEEK/MWCNT composites at temperatures below the melting point of PEEK did not affect the formation of MWCNT conductive network. This research may provide a new initiative for industry to explore and manufacture carbon-based composites with high-melting-temperature polymers. CNTs show a particular effect when selectively distributing in this kind of system. It can promote the dispersion of phase and also give conductivity to the blend at relatively small CNT levels. This may provide more useful ideas for the development of high-melting-temperature and antistatic or conductive materials.

## 2. Experimental

### 2.1. Materials

Poly(phenylene sulfide) (PPS-HB) was obtained from Deyang Science and Technology Company (Deyang, China) with relative density of 1.3 g·cm^–3^. Poly(ether ether ketone) (PEEK, 770PF) was purchased from Jilin Company (Suzhou, China) with a particle size smaller than 200 mesh. Carbon nanotubes (CNT) were obtained from Nanjing JC-Nano Tech Company (Nanjing, China) with an outer diameter of 10–30 nm and length of 10–30 μm.

### 2.2. Preparation of Samples

In this work, three methods were used to manufacture the composite samples: A, B, and C. Method A: All components (PPS, PEEK, and MWCNT) were homogenized and compounded in a SHJ20 twin-screw extruder at 360 °C to obtain PPS/PEEK/MWCNT composites. Method B: PPS was melt-mixed with PEEK in a twin-screw extruder at 360 °C, then the prepared blend was slowly cooled with cold air. In the end, the blend was melt-mixed with MWCNT in an internal mixer at 360 °C to obtain the PPS/PEEK/MWCNT composite. Method C: PPS was melt-mixed with PEEK in a twin-screw extruder at 360 °C, the extruder temperature range at the front was 355–365 °C, at the center was 360–365 °C, and at the die was 320–340 °C. The compounds were slowly cooled with cold air to room temperature. In the end, the blends were melt mixed with MWCNT in an internal mixer at 300 °C to obtain the PPS/PEEK/MWCNT composite.

To examine the process stability, the PPS/PEEK/MWCNT composite sample filled with 1 wt % MWCNT using method C was formed into pellet specimens using an injection molding machine for electrical conductivity measurements. The pellet specimens were broken into small pieces and, again, were processed into pellet specimens for conductivity measurements. The conductivity examinations were performed on pellet specimens to observe the effects of the repetition of molding times.

### 2.3. Characterization Techniques

#### 2.3.1. Contact Angle Examinations

A JJC-I (Changchun Optical Instrument Company, Changchun, China) wet contact angle goniometer was used to examine the contact angles. In this experiment, selected representative liquids were vertically added to the solid surface at a distance of approximately 3 mm from the solid surface. The liquid volume was 2–5 mm^3^ and the measurement time shorter than 1 min. At least 10 contact angles for each type of composite were measured to get an average value.

#### 2.3.2. Thermal Characterization

A Q200 differential scanning calorimeter (DSC, TA Instruments, New Castle, DE, USA) was used to study the thermal behavior of PPS/CNT composites. Samples of 8–9 mg were accurately weighed for DSC examinations. All measurements were performed in a nitrogen atmosphere.

To examine the non-isothermal crystallization and melting behavior of materials, a composite sample was rapidly heated to 380 °C and held for 5 min. Subsequently, it was cooled down to 60 °C at a cooling rate of 10 °C·min^−1^ for the crystallization behavior observations. Then, the same sample was reheated to 380 °C at 10 °C·min^−1^ heating rate for the melting behavior investigation.

#### 2.3.3. Measurement of Electrical Conductivity

A SRM-110 (Wolfgang Warmbier, Pinion Company, Hilzingen, Germany) surface resistance meter was used to examine the electrical conductivity of composite specimens. At least five specimens of each type of composite were examined and the average values were reported.

#### 2.3.4. Morphology Examination

The specimens that were broken in the impact examinations were used for morphology studies. The fracture surfaces of the specimens were sputter-coated with gold before conducting microscopic observations with a Philips XL-30 environmental scanning electron microscope (ESEM, Philips company, Eindhoven, The Netherlands) at an acceleration voltage of 20 kV.

## 3. Results and Discussion

### 3.1. Theoretical Calculation of Interface Affinity between MWCNT Particles and Blend Components

Generally, the selective distribution of MWCNTs particles in an immiscible polymer blend is defined by wetting coefficient ω_a_ [15]. PPS and PEEK are fully compatible in the melt [16] but they are separated in the solid-phase blending system [17]. Addition of MWCNT to the PPS/PEEK blend melt, a composition with strong molecular chain affinity, may express the priority adsorption at the surface of MWCNT particles due to different interface affinity of molecular weights of the two polymers. With the cooling and solidification process, MWCNT particles are wrapped into the strong affinity component phase. Therefore, ω_a_ can still be proposed to define the selective distribution of CNT particles with a relationship as described by Equation (1) [16]:

(1) $$\omega_{a}=\frac{\gamma_{CNTs-polymer1}-\gamma_{CNTs-polymer1}}{\gamma_{polymer1,2}}$$

where, γ*_CNTs-polymer_*_1_, γ*_CNTs-polymer_*_2_, and γ*_polymer_*_1,2_ are the interfacial tensions between MWCNT particles and polymers 1 and 2, and between the two polymers, respectively. When ω_a_ > 1, MWCNT particles would preferentially be located in polymer 2; when ω_a_ < 1, MWCNT particles preferentially distribute in polymer 1; and when 0 < ω_a_ < 1, MWCNT particles distribute at the interface between the two polymers.

The interfacial tension can be calculated according to the harmonic mean of Equation (2) and geometric mean of Equation (3) [18]:

(2) $$\gamma_{12}=\gamma_{1}+\gamma_{12}-4(\frac{\gamma_{1}^{d}\gamma_{2}^{d}}{\gamma_{1}^{d}+\gamma_{2}^{d}}+\frac{\gamma_{1}^{p}\gamma_{2}^{p}}{\gamma_{1}^{p}+\gamma_{2}^{p}})$$

(3) $$\gamma_{12}=\gamma_{1}+\gamma_{12}-2(\sqrt{\gamma_{1}^{d}\gamma_{2}^{d}}+\sqrt{\gamma_{1}^{p}\gamma_{2}^{p}})$$

In these equations, γ*_i_* is the surface tension of component *i*, and γ*_di_* and γ*_pi_* are the dispersive and polar parts of the surface tension for component *i*, respectively. According to the geometric mean method [19], the surface tension can be calculated using Equations (4) and (5):

(4) $$\gamma_{LV}(1+\cos\theta )=2(\sqrt{\gamma_{SV}^{d}\gamma_{LV}^{d}}+\sqrt{\gamma_{SV}^{p}\gamma_{LV}^{p}})$$

(5) $$\gamma_{SV}=\gamma_{\text{SV}}^{d}+\gamma_{SV}^{P}$$

where θ is the contact angle, subscripts “LV” and “SV” denote the interfacial liquid vapor and surface vapor tensions, respectively. The superscripts “d” and “p” denote the dispersion and polar components of the total surface tension, γ*_SV_*, respectively. The surface data of selected representative liquids, double-distilled water (H_2_O), and methylene diiodide (CH_2_I_2_) are listed in Table 1 [20].

**Table 1:** Surface tension data of selected examined liquids.

Liquid	(N·m^−1^)	(N·m^−1^)	(N·m^−1^)
H_2_O	72.8	21.8	51.0
CH_2_I_2_	50.8	48.5	2.3

In this work, the contact angles on the surfaces of PPS and PEEK were respectively measured as 90.38° and 66.21° for H_2_O, and 24.78° and 24.79° for CH_2_I_2_. The surface tensions were calculated using Equations (4) and (5). The results are presented in Table 2. For MWCNT particles, the corresponding surface tension data have been suggested as 27.8 mN·m^−1^ for γ, 17.6 mN·m^−1^ for γ^d^, and 10.2 mN·m^−1^ for γ^p^_v_ [21]. The interfacial tensions between components were then calculated with Equations (2) and (3). The results of calculations are presented in Table 3. Finally, ω_a_ data for MWCNTs introduced into PPS/PEEK blend were calculated as 1.5 (using the harmonic mean equation) and 2.4 (based on the geometric mean equation). MWCNT particles were predicted as being localized in the PEEK phase of the composite.

**Table 2:** Surface energy data for components of composites.

Components	γ at 20 °C (mN·m^−1^)	–dγ/dT (mN·m^−1^)	γ at 360 °C (mN·m^−1^)	γ^d^ at 360 °C (mN·m^−1^)	γ^p^ at 360 °C (mN·m^−1^)	χ^p^
PPS (25 °C)	46.99	0.070	21.08	20.92	0.16	0.0076
PEEK (25 °C)	48.87	0.073	21.93	17.80	4.13	0.19
MWCNTs	27.8	0	27.8	17.6	10.2	0.37

γ = γ^d^ + γ^p^; χ^p^ = γ^p^/γ; γ^d^, dispersive component of γ; γ^p^, polar component of γ; χ^p^, polarity; *T*, temperature.

**Table 3:** Interfacial tensions as calculated using harmonic and geometric mean equations.

Component Couple	Interfacial Tension	
	Harmonic Mean Equation (mn·m^−1^)	Geometric Mean Equation (mn·m^−1^)
PPS-PEEK	3.93	2.79
PPS-CNTs	10.02	7.95
PEEK-CNTs	4.16	1.35

In addition to the interfacial energy, blending process and viscosity are also considered as two important factors to determine the selective distribution of MWCNT particles in two-phase polymer blends systems [22]. The melt viscosities of blends determine the formation of the PPS continuous phase in a wide range of compositions [23] because of the 50 °C higher melting point of PEEK than that of PPS [17]. When CNT concentration is distributed in the PPS phase, the reduction of the seepage threshold may be achieved owing to the volume exclusion effect of PEEK.

### 3.2. Effects of Blending Process

**Figure 1:**
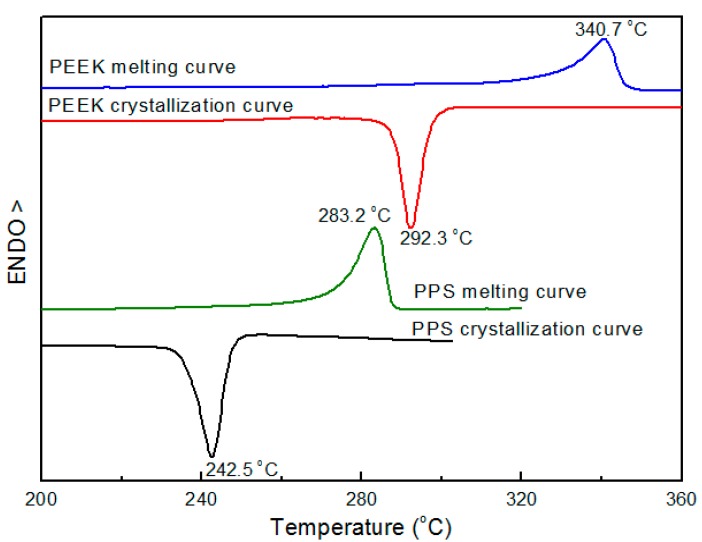
Crystallization and melting thermograms of neat PPS and PEEK.

From DSC examination results (Figure 1), melting (*T*_m_) and crystallization peak (*T*_c_) temperatures for PPS were observed at 283.2 and 242.5 °C, respectively. These temperatures for PEEK were determined at 340.7 and 292.3 °C, respectively. Therefore, when PEEK turned into a solid state at 292.3 to 283.2 °C, PPS was still in the melt state during the process of cooling the PPS/PEEK blend from the melt to room temperature.

**Figure 2:**
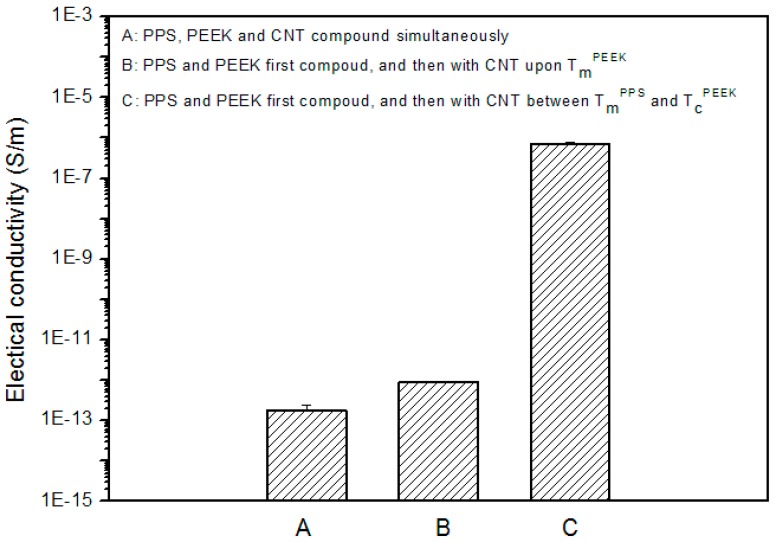
Electrical conductivity of PPS/PEEK/MWCNT nanocomposites containing 1 wt % MWCNT prepared according to methods A, B, and C.

**Figure 3:**
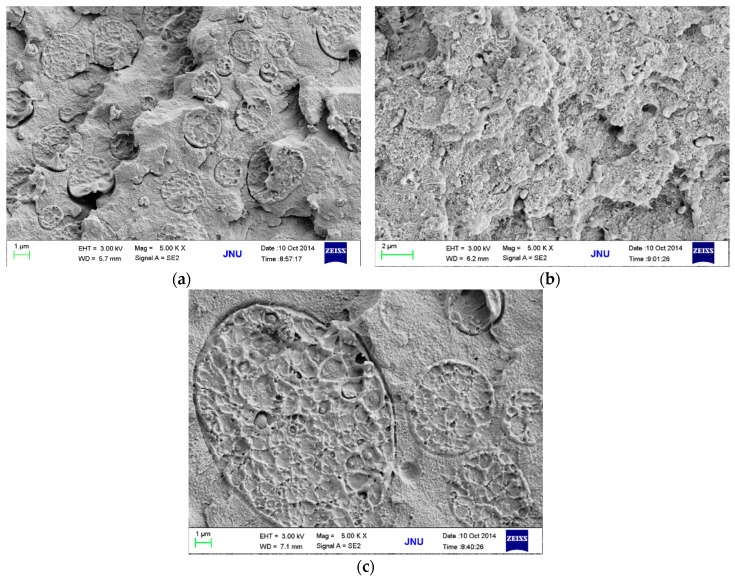
SEM micrographs of PPS/PEEK/MWCNT nanocomposites containing 1 wt % MWCNT prepared according to methods A (**a**), B (**b**), and C (**c**).

The above results provided us with a blending procedure (mainly refering to the mixing order and blending temperature) to obtain PPS/PEEK/MWCNT ternary nanocomposites with different MWCNT distribution patterns. To optimize the blending process, three different methods were used to prepare PPS/PEEK/MWCNT nanocomposite filled with 1 wt % MWCNT. Figure 2 and Figure 3 show the electrical conductivity values and the cross section SEM micrographs of the three nanocomposites. PPS/PEEK/MWCNT composites were prepared through blending of PPS, PEEK, and MWCNT components at the same time at 360 °C (method A). The conductivity values were observed at the same order of magnitude (10^−13^ S·m^−1^) with that of the PPS/PEEK blend, indicating that no conductive networks were formed in the composites. Previous papers have disclosed similar observations because of the distribution of MWCNT in two phases when the blending of raw materials is conducted simultaneously [17]. Even though particle distribution in the polymer matrix was uniform, the MWCNT content of 1 wt % still failed to form a conductive network in the whole blend matrix. Island structures were formed due to the solid phase separation of PEEK and PPS, which increased the insulating interfaces of the matrix (Figure 3a). PPS was melt-mixed with PEEK at 360 °C, and then was slowly cooled with cold air flow. At the end, the blend was melt-mixed at 360 °C with MWCNT to obtain a PPS/PEEK/MWCNT composite. The electrical conductivity values of composites prepared through the above method B was slightly larger than that of the composite manufactured through method A, but it was still at the same order of magnitude of 10^−13^ S·m^−1^. It can be assumed that during manufacture of composites, PEEK melted two times at temperatures above its melting point, which significantly decreases the size of the PEEK phase (Figure 3b), but MWCNT particles still distribute in the entire matrix. In method C, PPS was melt-mixed with PEEK at 360 °C and then slowly cooled with cold air flow. In the last stage, the blend was melt-mixed with MWCNT at 300 °C to obtain a PPS/PEEK/MWCNT composite. The conductivity values of composites prepared through method C reached 7.1 × 10^−7^ S·m^−1^, an increase of almost six orders of magnitude compared with that of composites prepared in methods A and B. Interestingly, what can be observed is a similar sausage structure in the SEM micrograph shown in Figure 3c. It seems that when PPS and PEEK are melt-mixed in the twin screw extruder and then are continuously cooled down, PEEK may transform rapidly into its solid state with insufficient time for all macromolecular chains of PPS to be separated from the PEEK phase. The complete solid phase separation induces the formation of sausage-type structures. Such structures are not destroyed in the second melt blending process at 300 °C, which forces MWCNT particles to be distributed only in the PPS matrix outside the similar sausage structures, resulting in the formation of conductive networks.

### 3.3. Effects of MWCNT Content on Electrical Conductivity and Morphology

**Figure 4:**
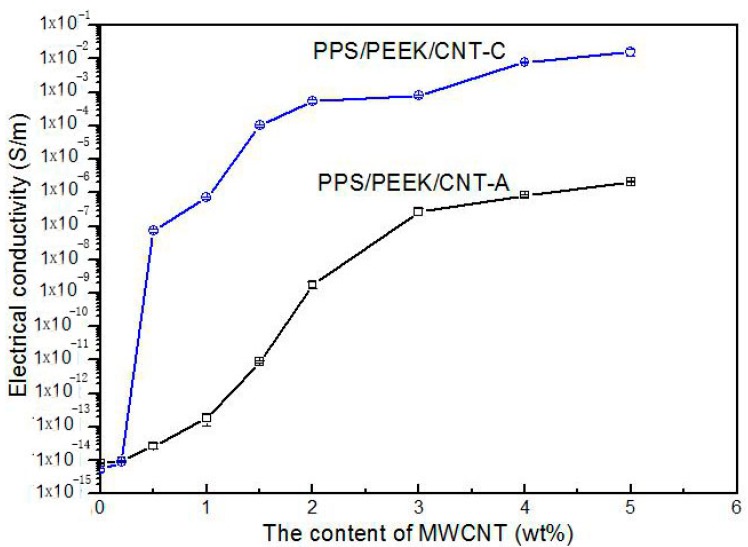
Variations of electrical conductivity of PPS/PEEK/MWCNT nanocomposites with different MWCNT contents prepared according to methods A and C.

**Figure 5:**
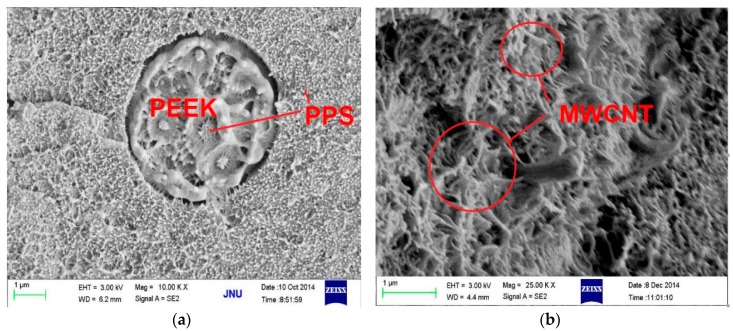
SEM micrographs of PPS/PEEK/MWCNT composites filled with 0.5 wt % MWCNT prepared via method C at two different magnifications. (**a**) 10,000×, and (**b**) 25,000×.

To explore the influence of similar sausage structures on the conductivity of PPS/PEEK/MWCNT nanocomposites, materials with various MWCNT contents were prepared using methods A and C. Figure 4 shows the variations of electrical conductivity of nanocomposites against MWCNT content prepared using the two methods. Obviously, the electrical conductivity of nanocomposites increased with increasing MWCNT content. It is interesting to note that, in this study, the electrical conductivity values of composites with all the MWCNT content prepared via method C were larger than those prepared using method A. This can be attributed to the even distribution of particles in the matrix in composites prepared with method A, whereas, in the case of using method C, the particles only distributed in the PPS phase and the actual MWCNT concentration was significantly larger than that in the other kinds of composite. When MWCNT content increased from 0.2 to 2 wt %, because of the volume exclusion effect of PEEK with a similar sausage structure, MWCNT particles were only distributed in the PPS continuous phase. This phenomenon caused a rapid increase of electrical conductivity of composite from 10^−13^ to 10^−3^ S·m^−1^. With a further increase of MWCNT content, the electrical conductivity of the composite changed slowly, indicating the formation of a conductive network when 2 wt % of MWCNTs was added to the polymer blend. A similar phenomenon was observed by Qi [24]. Micrographs in Figure 5a,b show morphology of PPS/PEEK/MWCNT composites filled with 0.5 wt % MWCNT prepared via method C at two different magnifications. They clearly reveal that PPS forms the continuous matrix phase and PEEK, the dispersed phase, whereas MWCNT particles are only distributed in the PPS continuous phase. MWCNT particles (white dots) are evenly dispersed in the matrix with no evidence of reunion. Most of the MWCNT particles are evenly spread out, which are electrically conductive, forming the network of particles in contact with each other. In addition, the fuzzy interface between PPS and MWCNT particles suggests good interface bonding between them. To estimate the percolation threshold concentration, the experimental data were fitted using the power law equation (Equations (6) and (7)) for the composites’ conductivities near the percolation threshold [25]:

σ_DC_ ∝ (*p* – *p*_c_)*^t^* for *p* > *p*_c_ (6)

σ_DC_ ∝ (*p* – *p*_c_)^−s^ for *p* > *p*_c_ (7)

where σ_DC_ is the conductivity of the composite with various filler contents, *p* is the mass fraction of the filler, *p*_c_ is EPT value, and *t* is the critical exponent related to the dimensionality of the percolated network associated with dimensionality of the percolated network ranging between 1.1 and 1.3 for 2D networks [26], and between 1.6 and 2.0 for 3D networks [27].

**Figure 6:**
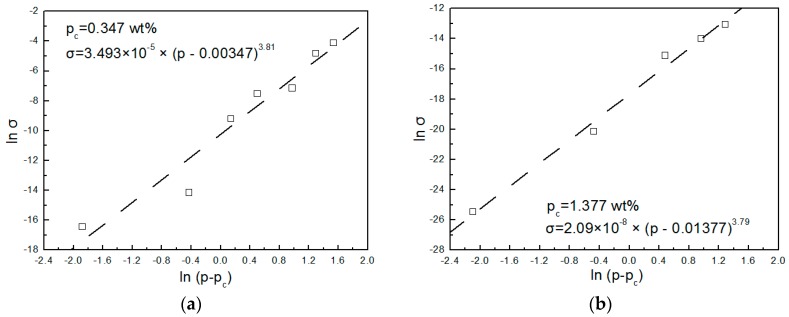
Best linear curve fitting for PPS/PEEK/MWCNT nanocomposites with different MWCNT contents prepared according to methods A (**a**) and C (**b**).

To assess the EPT value, the electrical conductivity values of PPS/PEEK/MWCNT nanocomposites prepared via methods A and C were calculated using the EPT scaling law [28]. In the calculations, we found EPT values of 0.347 and 1.377 wt %, and critical index t of 3.81 and 3.79 for methods A and C, respectively. The standard deviations of 0.43 and 0.22 were found for the two methods A and C, respectively. The best linear curve fittings for the EPT are shown in Figure 6a,b. The large calculated t is different from that predicted from the normalization theory of three dimensional percolation systems. Such a high t value has been discussed in detail in many previous publications, including examples and reasons for this contradiction [29], such as that between the index value and the predicted values of PS/PMMA/MWCNT [30] and of PP/MWCNT composites [31]. In this article, we are not discussing this matter.

### 3.4. Stability of Products and Repetition of Processing

**Figure 7:**
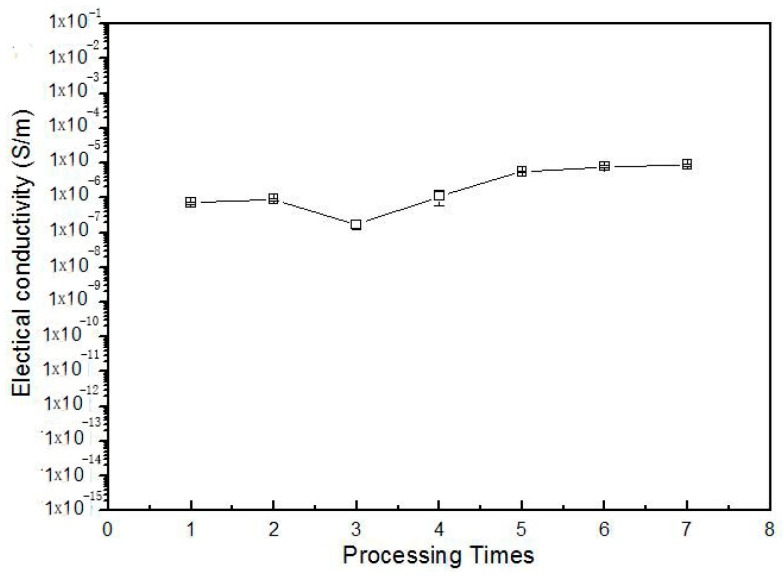
Variation of electrical conductivity for PPS/PEEK/MWCNT nanocomposites containing 1 wt % MWCNT prepared via methods C with repetition of the injection molding process.

**Figure 8:**
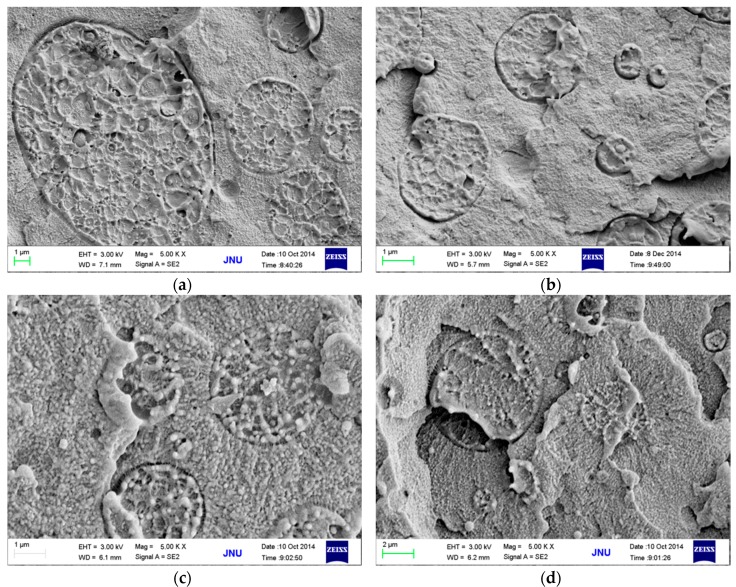
SEM micrographs of samples molded for once (**a**); three times (**b**); six times (**c**); and seven times (**d**).

In this work, we prepared composites using method C molded only at the temperatures below melting point of PEEK, different from previous studies. It can be expected that such conditions will not destroy the similar sausage structure of PEEK phase and the heat flow occurs only in the PPS phase, thus, the material displays good shape stability and steady electrical properties. To verify the above hypothesizes, the PPS/PEEK/MWCNT composite filled with 1 wt % MWCNT was prepared via method C and injection molded seven times with a conventional injection molding machine. The electrical conductivity values of samples experiencing various molding times are plotted in Figure 7. The observations indicate that, in general, with the increase of the process times, the conductivity of the composite slightly increases. However, it can be observed from Figure 8a,b that the large similar sausage structure in composite was broken into small structures, making the size distribution of similar sausage structures narrower, resulting a smaller electrical conductivity after the sample was molded for the third time. Micrographs in Figure 8c,d indicate that after six and seven times processing, the size of the sausage phase was continually decreasing, resulting in an increase in conductivity. This observation fully confirms the original hypothesis of this article that a stable and practical electrically-conductive nanocomposite may be prepared at different processing temperatures.

## 4. Conclusions

In summary, based on the control of the blending plan, conductive PPS/PEEK/MWCNT nanocomposites were prepared through a step-melt blending method. Firstly, PPS and PEEK were completely melted and blended. Blends of PPS and PEEK with similar sausage structures of PEEK as a dispersed phase were prepared. Then, PPS was blended with MWCNTs at the melting temperature of PPS. The MWCNT particles tend to disperse in the PPS melt with low viscosity; thus, MWCNT particles are concentrated in the PPS continuous phase of PPS/PEEK/MWCNT nanocomposites. MWCNT provides PPS/PEEK blend with electrical conductivity. The calculated EPT value for the composite decreased to 0.347 wt % due to the volume exclusion effect of similar sausage structures. The electrical conductivity of composite filled with 0.5 wt % of MWCNT was measured as 7.3 × 10^−8^ S·m^−1^. The prepared nanocomposites remained consistent with high conductivity even when they were repeatedly processed in injection molding. Our observations confirm that the two-phase compatible blend system may also realize double percolation with low EPT, which provides a new idea for the preparation of practical multiphase polymer conductive materials.
